# Synthesis of single-phase *L*1_0_-FeNi magnet powder by nitrogen insertion and topotactic extraction

**DOI:** 10.1038/s41598-017-13562-2

**Published:** 2017-10-16

**Authors:** Sho Goto, Hiroaki Kura, Eiji Watanabe, Yasushi Hayashi, Hideto Yanagihara, Yusuke Shimada, Masaki Mizuguchi, Koki Takanashi, Eiji Kita

**Affiliations:** 1 Advanced Research and Innovation Center, DENSO Corporation, Aichi, 470–0111 Japan; 2 Institute of Applied Physics, University of Tsukuba, Ibaraki, 305–8573 Japan; 3 Institute for Materials Research, Tohoku University, Sendai, 980–8577 Japan; 4 National Institute of Technology, Ibaraki College, Ibaraki, 312–8508 Japan

## Abstract

Tetrataenite (*L*1_0_-FeNi) is a promising candidate for use as a permanent magnet free of rare-earth elements because of its favorable properties. In this study, single-phase *L*1_0_-FeNi powder with a high degree of order was synthesized through a new method, nitrogen insertion and topotactic extraction (NITE). In the method, FeNiN, which has the same ordered arrangement as *L*1_0_-FeNi, is formed by nitriding A1-FeNi powder with ammonia gas. Subsequently, FeNiN is denitrided by topotactic reaction to derive single-phase *L*1_0_-FeNi with an order parameter of 0.71. The transformation of disordered-phase FeNi into the *L*1_0_ phase increased the coercive force from 14.5 kA/m to 142 kA/m. The proposed method not only significantly accelerates the development of magnets using *L*1_0_-FeNi but also offers a new synthesis route to obtain ordered alloys in non-equilibrium states.

## Introduction

In recent years, there has been an increased demand for highly functional magnetic materials in a variety of fields, including the automotive industry, electrical and electronics industry, medicine, and environmental science, in which potential applications include wind power generation. Alternatives for rare-earth magnets such as Nd-Fe-B and Sm-Fe-N, which are known as high-performance magnets, are required because of the low thermal resistance of these magnets and the risk of depleting resources^1^. *L*1_0_-FeNi is drawing much attention as a magnet free of rare-earth elements. Minute quantities of *L*1_0_-FeNi are contained in iron meteorites^2,3^, and although it is comprised of common elements such as Fe and Ni, it has high uniaxial magnetic anisotropy (>1 × 10^6^ J/m^3^)^4,5^. The saturation magnetic flux density of *L*1_0_-FeNi is 1.6 T (154 A∙m^2^/kg), which is comparable to that of Nd-Fe-B. The Curie point is 550 °C or higher, which is higher than that of conventional magnets^6^. Therefore, *L*1_0_-FeNi is expected to be applied as a permanent magnet, and studies are being conducted to evaluate its fundamental properties and to develop artificial synthesis methods^4,5,7–12^.

**Figure 1:**
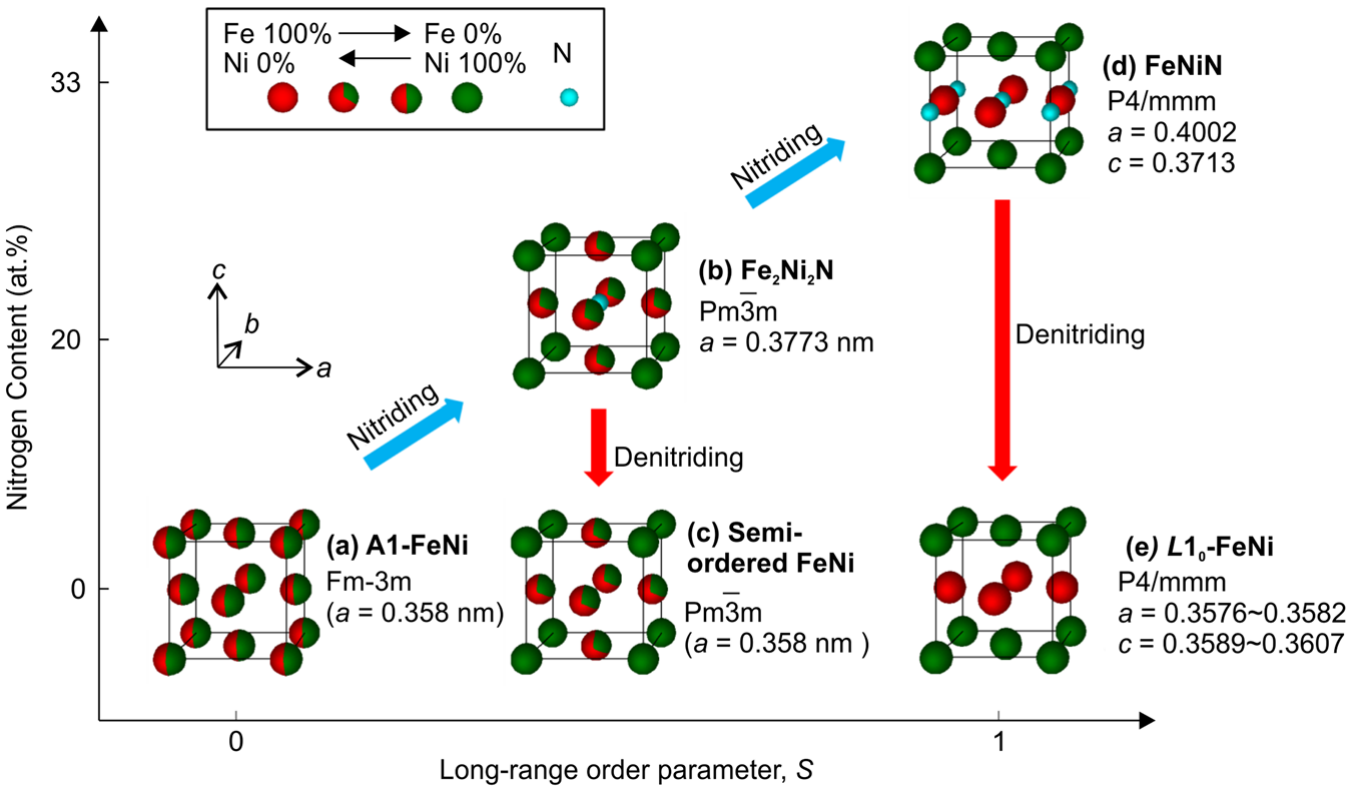
Conceptual diagram for fabrication path of ordered FeNi alloys by NITE method Models of crystal lattices for (**a**) A1-FeNi, (**b**) Fe2Ni2N^17,18^, (**c**) semi-ordered FeNi, (**d**) FeNiN^18^, and (**e**) *L*1_0_-FeNi^3^ are depicted with Fe (red), Ni (green), and N (light blue) atoms. Atoms identified by red and green indicate that Fe and Ni are randomly arranged according to the ratios of coloured areas. Face-centred cubic (FCC) structures are depicted as a basis for comparison, although unit cells are individually different. The lattice constants in (**b**), (**d**), and (**e**) are taken from literature data, while lattice constants in (**a**) and (**c**) are values derived in this research.

*L*1_0_-FeNi is an ordered alloy containing equiatomic Fe and Ni, as shown in Fig. 1e, in which atomic layers of Fe and Ni are stacked alternatively along the c-axis of the FCT crystal structure. Its easy axis of magnetization corresponds to the c-axis. The magnetic anisotropy constant (*K*_u_) of *L*1_0_-FeNi is strongly correlated with the long-range order parameter, *S*, which is defined as follows:

1 $$S=2{p}_{Fe}-1,$$

where *p*_Fe_ is the Fe occupancy at the Fe site. In order to use *L*1_0_-FeNi as a permanent magnet, the formation of high-*S L*1_0_-FeNi is essential^13^ so that the magnetic anisotropy of *L*1_0_-FeNi is maximized. Furthermore, it is essential to derive single-phase *L*1_0_-FeNi as bulk or bulkable powder. Artificially synthesized *L*1_0_–FeNi bulk materials reported thus far have a low *S* and/or low abundance ratio^9–12^. The extremely low stability of the *L*1_0_ structure prevents the formation of high-*S*, single-phase bulk *L*1_0_–FeNi. Therefore, an effective means to achieve an *L*1_0_-FeNi magnet is yet to be developed.

The disorder-order transition is generally a thermally activated process, and ordered alloys are derived by heat-treating the disordered alloy at temperatures less than or equal to the order-disorder transition temperature, *T*_λ_. The *T*_λ_ of *L*1_0_-FeNi has been estimated to range in 200–320 °C^14–16^. Therefore, heat treatment must be performed at a temperature sufficiently lower than this range in order to derive high-*S L*1_0_-FeNi. However, since atomic diffusion is extremely slow at such a temperature, an astronomical amount of time would be required to synthesize *L*1_0_-FeNi. This indicates that high-*S L*1_0_-FeNi cannot be derived through the conventional quasi-equilibrium process that promotes the formation of *L*1_0_ through a mutual diffusion of Fe and Ni and by utilizing the stability of *L*1_0_-FeNi as the driving force for ordering.

Taking the above into consideration, we considered developing an ordering method that does not rely on the low stability of *L*1_0_-FeNi to be the driving force. In this study, we developed an ordering method called nitrogen insertion and topotactic extraction (NITE), which is an ordered-alloy formation process involving a stable ordered intermediate material. In this method, the ordered arrangement of FeNi with nitriding as the trigger is combined with topotactic denitriding for extracting nitrogen atoms from FeNi nitrides without damaging the crystal structure. The proposed method is significantly different from the conventional thermally activated process in that the ordered alloys can be derived directly by denitriding.

The schematic for synthesizing ordered alloys using the NITE method is shown in Fig. 1. Nitriding the FeNi random alloy (Al-FeNi), as shown in Fig. 1a, results in the insertion of nitrogen atoms in the body-center positions (1/2 1/2 1/2), with body-corner positions occupied by nickel, to form Fe_2_Ni_2_N (Fig. 1b)^17,18^. Low-*S* FeNi ordered alloy is derived with the topotactic denitriding of Fe_2_Ni_2_N, as shown in Fig. 1c. Further nitriding of Fe_2_Ni_2_N results in the insertion of nitrogen in (0 0 1/2), with the (001) plane occupied by nickel and the (002) plane occupied by iron, to derive FeNiN (Fig. 1d)^18^. High-*S L*1_0_-FeNi is derived by the topotactic denitriding of FeNiN because the metallic atom arrangement of FeNiN is identical to that of *L*1_0_-FeNi (Fig. 1e).

Special experimental facilities such as the atomic furnace for neutron irradiation^4,5^ are no longer required, because the NITE method implements nitriding and denitriding with an electric furnace in which ammonia or hydrogen gas can be introduced. The NITE method is expected to accelerate magnet development dramatically because high-S *L*1_0_-FeNi can be derived in a single phase, despite the simplicity of this method. The formation process of ordered *L*1_0_-FeNi alloys by the NITE method is discussed hereafter, with focus on the changes that occur in crystals in the processes of nitriding by heat-treating FeNi powder samples in ammonia gas and denitriding by heat-treating in hydrogen gas. The formation of high-S and single-phase *L*1_0_-FeNi magnetic powder is verified based on the results of element mapping using scanning transmission electron microscopy (STEM) and the results of magnetometry.

## Result

**Figure 2:**
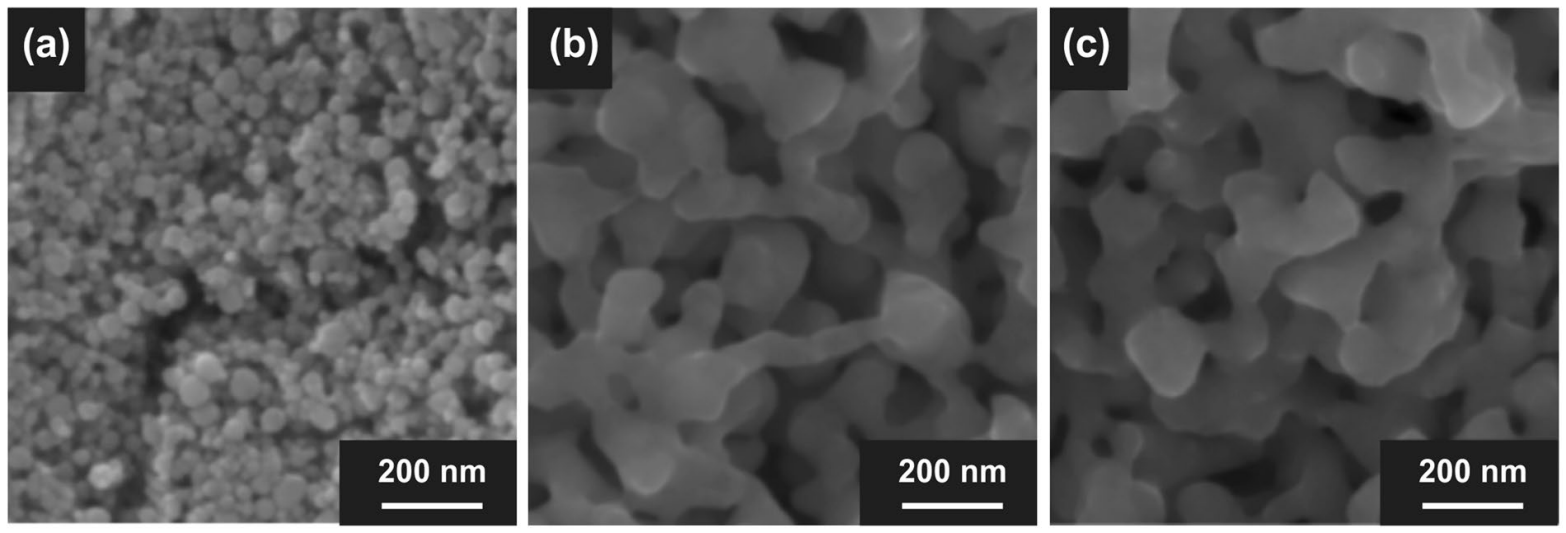
Morphological changes of FeNi powder through the NITE process. SEM images of (**a**) FeNi powder starting material fabricated through the thermal plasma method; (**b**) FeNi powder after removal of oxidized film; (**c**) FeNi powder after NITE processing. Sintering, growth, and necking of particles occurred during the removal of the oxidized film. No changes in particle shapes were observed after the NITE process.

FeNi nanoparticles with an average diameter of 44 nm, fabricated using the thermal plasma method^19^, were selected as the starting material to accelerate reactions during the NITE process. An oxide film exists on the surface of FeNi nanoparticles (as shown in Supplementary Figure 1), which inhibits nitriding. Therefore, hydrogen reduction was performed as pretreatment to reduce or remove the oxide film, and the NITE process was performed on the resulting FeNi. Typical scanning electron microscope (SEM) images of the starting material before and after the pretreatment, which involved heat treatment in hydrogen (pre-FeNi), as well as an SEM image of FeNi powder after the NITE process, are shown in Fig. 2a–c (size distributions are shown in Supplementary Figure 2). Growth and necking occurred in these particles because of sintering during pretreatment. The mode diameter after sintering was approximately 90 nm. Hardly any changes occurred in the particle diameter during the NITE process that was performed subsequently, and sintering did not appear to have progressed further.

**Figure 3:**
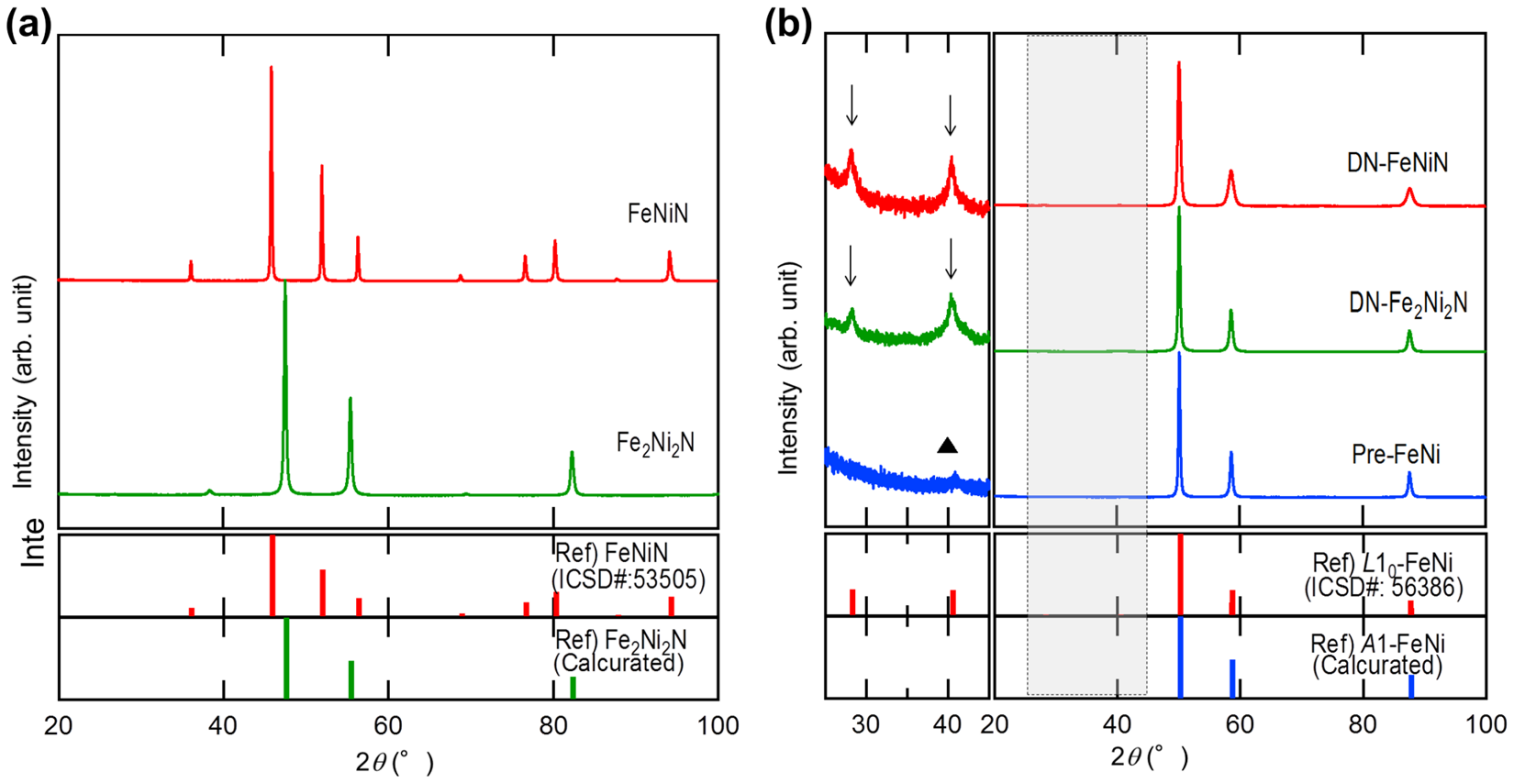
Crystal-structure changes of FeNi powder obtained using the NITE process. (**a**) X-ray patterns of FeNi nitride powder. Fe_2_Ni_2_N and FeNiN were derived in a single phase. (**b**) X-ray patterns of pre-FeNi, DN-Fe_2_Ni_2_N, and DN-FeNiN powders. The profiles of Fe_2_Ni_2_N estimated based on the Rietveld simulation and the literature data of FeNiN (Inorganic Crystal Structure Database Collection Code [ICSD#]: 53505) are shown in the lower segment of the same figure for comparison. Superlattice diffraction lines indicated by arrows were observed in addition to basic regression lines of A1-FeNi in DN-Fe_2_Ni_2_N and DN-FeNiN. The peak indicated with a triangle is believed to have been due to Ni_x_Fe_3-x_O_4_ generated by surface oxidation.

XRD powder patterns of Fe_2_Ni_2_N and FeNiN derived by the ammonia nitriding of FeNi are shown in Fig. 3a. Fe_2_Ni_2_N and FeNiN matched quite well with patterns based on calculation values or literature data, as evident from Fig. 3a. This indicates that FeNi nitrides have been derived in a single phase with a high degree of order. XRD powder patterns of pre-FeNi and FeNi powder derived by denitriding Fe_2_Ni_2_N (DN-Fe_2_Ni_2_N), as well as FeNi powder derived by denitriding FeNiN (DN-FeNiN), are shown in Fig. 3b. The literature data for *L*1_0_-FeNi with S = 1 (ICSD#: 56386) and calculated data for A1-FeNi are indicated in the lower part of the figure for comparison. The profiles in the diagram to the left in Fig. 3b show the diffracted intensity for the shaded portion in the diagram to the right, magnified by a factor of 50, to distinguish the superlattice diffraction lines. FeNi was evidently converted into an ordered alloy because the superlattice diffraction lines are clearly observed, as indicated by the arrows in the figure for DN-Fe_2_Ni_2_N and DN-FeNiN. Furthermore, all diffraction lines from the nitrides have disappeared in these profiles, with the lattice constant equal to that of the starting raw material, which was a = 0.358 nm. These results indicate that the nitrogen had completely detached from the materials. A broad peak was observed for pre-FeNi near 2θ = 41°, which coincided with the emergence positions of the diffraction peaks on the Miller index (311) plane of Ni_x_Fe_3-x_O_4_. This oxide is believed to have been formed from a reaction with oxygen in the atmosphere. Similar peaks overlapping with superlattice diffraction peaks in DN-Fe_2_Ni_2_N and DN-FeNiN were also observed.

The emergence angles of the superlattice diffraction peaks of DN-Fe_2_Ni_2_N and DN-FeNiN coincide. Therefore, these cannot be distinguished by sight. Supplementary Table 1 in the supplemental information lists the Miller index, inter-planar spacing, emergence angle, and normalized intensity of perfectly ordered *L*1_0_-FeNi and semi-ordered FeNi, as shown in Fig. 1(c), which were calculated by RIETAN-FP under the assumption that both lattice constants are 0.358 nm and the wavelength of X-rays is 0.1757 nm. DN-FeNiN with fixed sites of Fe and Ni is predicted to have a higher *S* than DN-Fe_2_Ni_2_N in the calculation, as shown in Fig. 1. The *S* of *L*1_0_-FeNi can be calculated using the (001) superlattice diffraction lines and the (111) integrated intensity ratio of basic diffraction lines $({I}_{(001)}/{I}_{(111)})$ as follows:

2 $$S\,=\sqrt{\frac{{({I}_{(001)}/{I}_{(111)})}^{obs}\,}{{({I}_{(001)}^{L{1}_{0}}/{I}_{(111)}^{L{1}_{0}})}^{cal}}.}$$

Here, the denominator is the integrated intensity ratio for *L*1_0_-FeNi with *S* = 1, derived by calculation. The numerator is the integrated intensity ratio derived experimentally from the XRD results. The *S* values of FeNi ordered alloys derived by the complete topotactic denitriding of ideal Fe_2_Ni_2_N and FeNiN, as shown in Fig. 1c,e, were 0.56 and 1.0, respectively. The *S* of DN-Fe_2_Ni_2_N and DN-FeNiN derived experimentally, on the other hand, were 0.53 and 0.71, respectively, according to Equation (2). The reason why the experimental value of *S* is lower than the ideal value is concerned that disordering of *L*1_0_ was occurred partially due to mutual diffusion of Fe and Ni during denitriding. However, the atomic arrangement of nitrides is presumed to have been passed on after denitriding because higher *S* was obtained using FeNiN as precursor than using Fe_2_Ni_2_N. *S* = 0.71 is the highest reported value for *L*1_0_-FeNi thus far. In the conventional processes in which ordering occurs by atomic diffusion, there is a limit to increasing the value of *S* due to issues such as the lagging atomic diffusion and low regularity of equilibrium states, whereas with the NITE method, there are no such restrictions, because ordered alloys are directly acquired from nitrides with a high degree of order. *L*1_0_-FeNi with a high *S* is believed to have been derived as a result. It is reasonable to assume that the FeNi ordered alloys were derived in a single phase because the nitrides were derived in a single phase, although *L*1_0_-FeNi in a meteorite or that synthesized by the conventional method was derived as a mixture with disordered FeNi which comprised the majority of the materials. Therefore, NITE is an effective method to achieve single-phase *L*1_0_-FeNi with a high degree of order.

**Figure 4:**
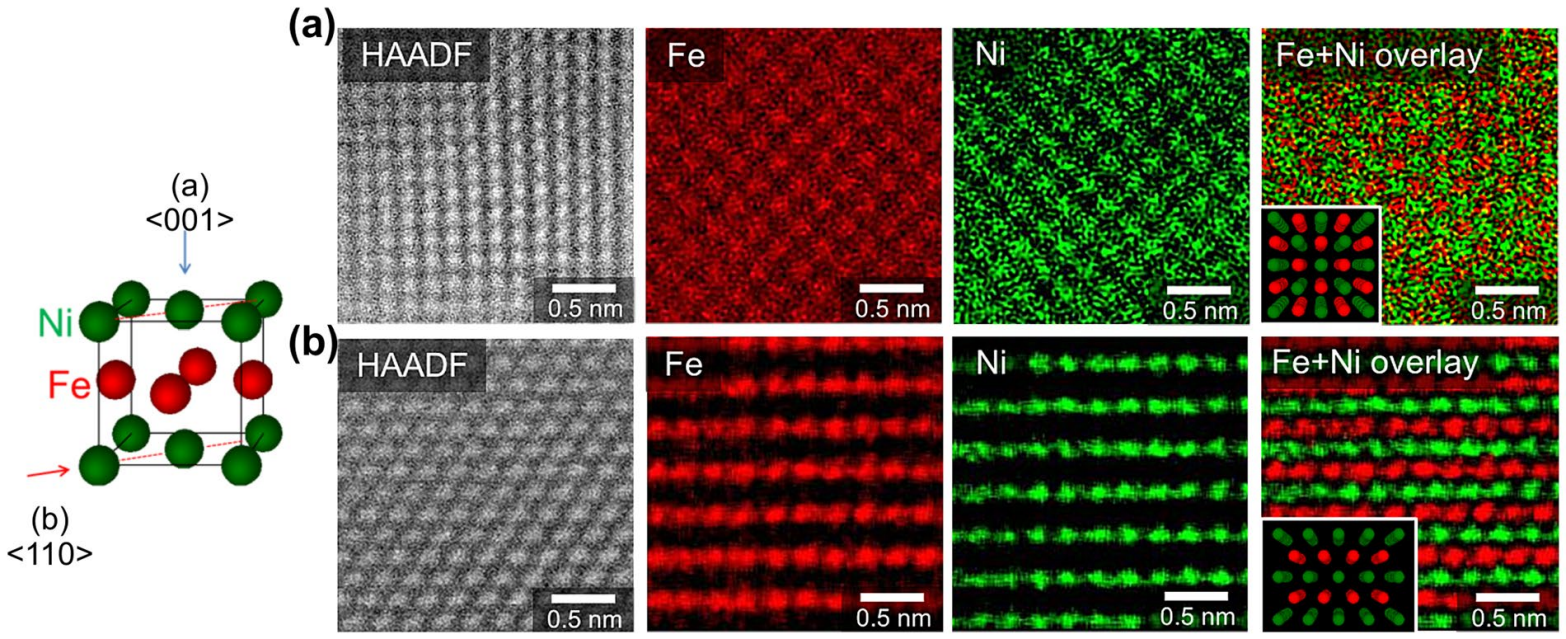
Direct observations on ordered states of DN-FeNiN. Results of HAADF imaging of DN-FeNiN, Fe element mapping, Ni element mapping, and element mapping overlay for Fe and Ni, observed from two directions using the atomic-resolution analytical STEM. The insets of the overlay images show CG images of lattices observed from < 001 > for (**a**) and from < 011 > for (**b**) with respect to *L*1_0_-FeNi.

Figure 4 shows the results of element mapping using STEM energy dispersive spectroscopy (STEM-EDS) with atomic resolution that clarify the microscopic ordered state of DN-FeNiN. Evaluations were conducted from two different perspectives with different crystal orientations. The insets of the overlapped images in Fig. 4a,b indicate the computer graphics (CG) images of lattices when *L*1_0_-FeNi with S = 1 was observed in the [001] and [110] directions. The mapping images for Fe and Ni from the two observation directions match each other extremely well, clarifying that DN-FeNiN is in the *L*1_0_ phase. Topotactic denitriding is the key for the NITE method owing to the reasons described above, and the formation of ordered alloys with a scheme that is completely different from conventional methods is possible with the passing on of the atomic arrangement of nitrides after denitriding. This constitutes the first successful visualization of the formation of *L*1_0_-FeNi. The separate evaluation of elements with STEM-EDS is believed to have been enabled by the high *S* of *L*1_0_-FeNi derived with the NITE method.

**Figure 5:**
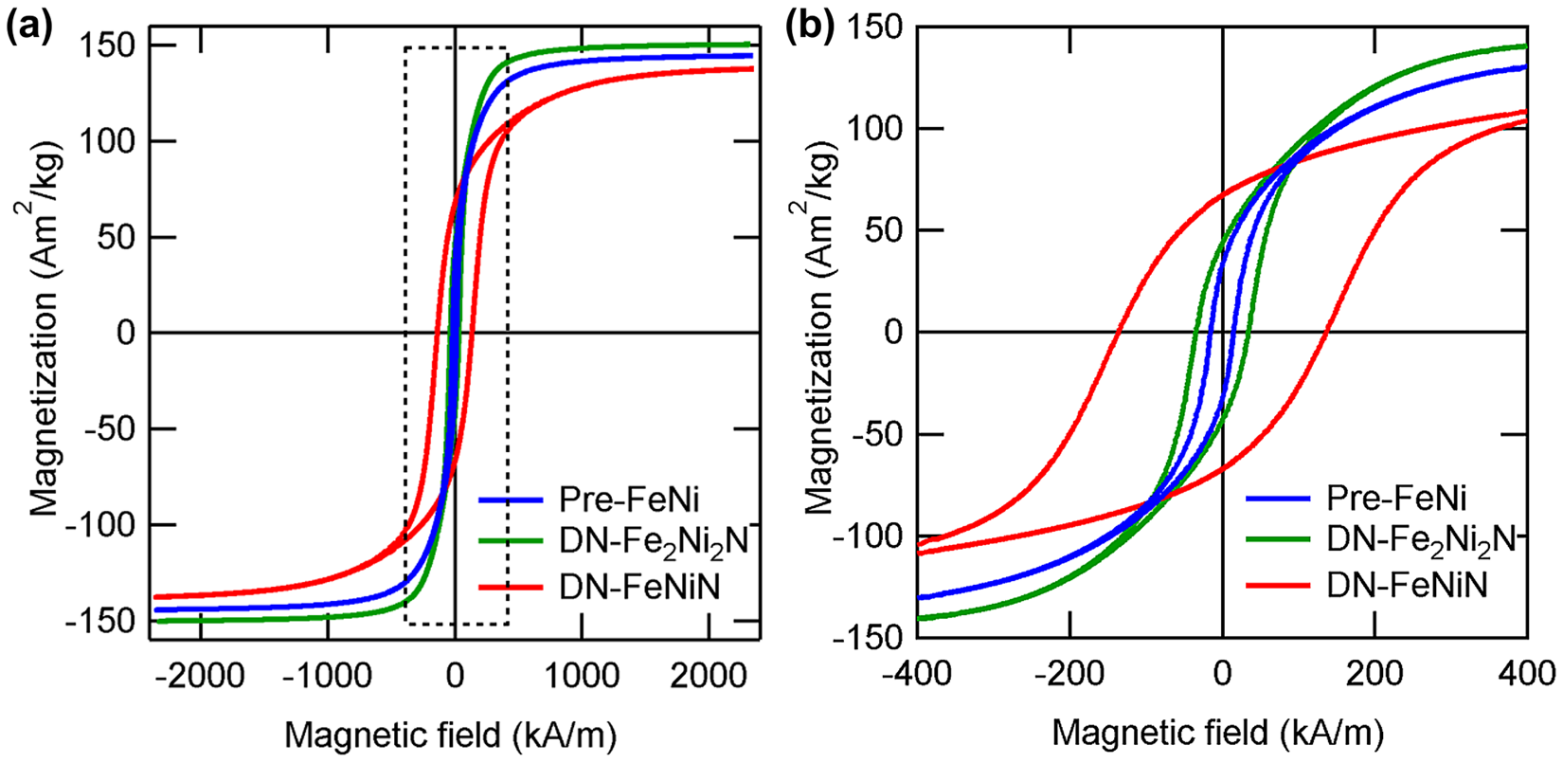
Comparison of Hysteresis curve of NITE processed FeNi powder (**a**) Hysteresis curves of pre-FeNi, DN-Fe_2_Ni_2_N, and DN-FeNiN. (**b**) Magnified hysteresis curve for the area indicated by the dotted rectangle in Fig. 5a.

The magnetic properties of FeNi are predicted to have changed significantly by the formation of an ordered structure. The M–H curves of pre-FeNi, DN-Fe_2_Ni_2_N, and DN-FeNiN at 300 K measured using a vibrating sample magnetometer (VSM) are shown in Fig. 5. All the plots are based on data corrected for the demagnetizing field with background removed. The magnetization *M*_H=2.4M_ under an applied magnetic field of 2.4 MA/m was 144, 151, and 139 Am^2^/kg for pre-FeNi, DN-Fe_2_Ni_2_N, and DN-FeNiN. The saturation magnetization of *L*1_0_-FeNi was estimated to be 154 Am^2^/kg in a past report^5^, compared to which the value of DN-FeNiN in the *L*1_0_ phase was smaller by approximately 10%. The reasons for this decrease in saturation magnetization include the reduction of ferromagnetic components due to the oxidation of particle surfaces and the improvement of magnetic anisotropy associated with the formation of the *L*1_0_ phase, which made the saturation of magnetization more difficult to achieve. A comparison of the coercive force *H*_c_ indicated that the *H*_c_ of pre-FeNi was 14.5 kA/m. The magneto-crystalline anisotropy and coercive force were low for pre-FeNi because it was in the A1 phase. The *H*_c_ of DN-Fe_2_Ni_2_N was 32.0 kA/m. DN-Fe_2_Ni_2_N is believed to have the crystal structure shown in Fig. 1c, which contains space groups equivalent to those of *L*1_2_ ordered alloys (Supplementary Figure 3 shows element mapping images of DN-Fe_2_Ni_2_N to support the above discussion). The *L*1_2_ structure is generally known to have low magnetic anisotropy, and examples for such cases include *L*1_2_-Fe_3_Pt^20^. DN-Fe_2_Ni_2_N is believed to have demonstrated a lower coercive force for the same reason. The *H*_c_ of DN-FeNiN was 142 kA/m. The magnetic anisotropy improved with the formation of *L*1_0_-FeNi, and *H*_c_ increased by a factor of approximately ten in comparison with that of pre-FeNi.

*L*1_0_-FeNi fabricated with the NITE method showed a high coercive force, as described above, and magnetization did not reach saturation even under an applied magnetic field of 2.4 MA/m. This suggests an extremely high magnetic anisotropy of *L*1_0_-FeNi. For comparison, the magnetization along the hard axis of magnetization of *L*1_0_-FeNi derived by heat treatment under neutron irradiation achieved saturation at approximately 1 MA/m^4,5^. *L*1_0_-FeNi derived from the NITE method clearly has a higher *K*_u_ than those reported in the past. The source of this higher *K*_u_ is evidently the high *S*. The *K*_u_ of *L*1_0_-type ordered alloys is known to increase in proportion to *S* to the power of 1.6–2.4^13,21^. In previous work, the *K*_u_ values of *L*1_0_-FeNi with *S* = 0.41 in a bulk and with *S* = 0.5 in a thin film were estimated at 1.37 × 10^6^ J/m^3^ ^5^ and 0.7 × 10^6^ J/m^3^ ^21^, respectively. The *K*_u_ of *L*1_0_-FeNi derived by the NITE method could not be estimated quantitatively because of sintering between particles, as shown in Fig. 2c; a single-crystal bulk or film is required to evaluate *K*_u_. However, the *S* of *L*1_0_-FeNi derived by the NITE method was 0.71, and when the aforementioned relationship between *S* and *K*_u_ is reflected, the *K*_u_ of *L*1_0_-FeNi is expected to reach 1.4 × 10^6^ J/m^3^ to 3 × 10^6^ J/m^3^. While this *K*_u_ is less than that of Nd-Fe-B (4.9 × 10^6^ J/m^3^), it is still an extremely high value considering that the material is free of rare-earth elements.

There are several issues, as described below, hampering the practical implementation of *L*1_0_-FeNi. Although high-*S L*1_0_-FeNi has high potential as a magnet, the manifestation of coercive force anticipated from the essential potential of the substance has not been achieved owing to sintering between particles, as shown in Fig. 2c, and the non-oriented crystal structure. For application as a permanent magnet, it is vital to improve *H*_c_ and the residual magnetization by magnetically isolating *L*1_0_-FeNi particles and orienting them along the easy axis of magnetization. The *S* of *L*1_0_-FeNi prepared in this study was 0.71. There is still room for further improvement of *K*_u_ by optimizing the topotactic denitriding reaction. If *S* can be increased from 0.71 to 1.0, an improvement of magnetic orientation by a factor of approximately two can be expected along with a *K*_u_ value comparable to that of rare-earth magnets. In future work, denitriding conditions preventing from reducing of S will be investigated through elucidation of a mechanism of the topotactic denitriding process. On the other hand, the *L*1_0_-FeNi synthesized in this study has sufficient thermal stability for practical applications because once high-S *L*1_0_-FeNi is formed by the NITE method, diffusion would be extremely slow at lower temperatures, resulting in a stable high-*S* phase. *L*1_0_-FeNi fabricated with the NITE method sustained an ordered structure up to 400 °C (as indicated in Supplementary Figure 4), but a transformation to a disordered phase occurred once the temperature reached 450 °C. It was reported that the temperature required for the progression of disordering in *L*1_0_-FeNi extracted from meteorites is 480 °C or higher^6^. There is a slight dependence of the disordering temperature on the material form. Furthermore, to fabricate high-performance *L*1_0_-FeNi magnets, a method for molding at or below the disordering temperatures is essential.

In summary, we proposed the NITE method and demonstrated that it is an effective method for fabricating *L*1_0_-FeN. The formation of an ordered phase through non-equilibrium processes in the NITE method might be extended for increasing the degree of order for ordered alloys other than FeNi. The NITE method has much potential for deriving ordered alloys that cannot possibly exist under equilibrium conditions, e.g., through the formation of the *L*1_2_ phase through the topotactic detachment of elements other than nitrogen (such as C or B). We hope that, in the future, the NITE method will be developed further to facilitate the derivation of completely new ordered alloys that are superior in terms of characteristics such as magnetism, toughness, and catalytic performance.

## Methods

### Synthesis

FeNi nanoparticles synthesized by the thermal plasma method, having an average diameter of 30 nm, were purchased from Nisshin Engineering and used as the starting material. Reduction by hydrogen and nitriding by ammonia were performed using a box-type electric furnace, which enabled the introduction of the aforementioned gases. The purity of the hydrogen gas and ammonia gas was 99.9999% or higher and 99.999% or higher, respectively. Typical processing conditions of the NITE method are described below.

1 g of FeNi nanoparticle as purchased was placed in the center of the furnace. Oxides that formed on the specimen surfaces immediately after purchase were reduced by heat treatment at 400 °C in hydrogen gas flowing at a rate of 1 L/min for 2 h. Nitrided FeNi was then obtained by nitriding the specimen at 300 °C in a large amount of ammonia flowing at 5 L/min. Fe_2_Ni_2_N and FeNiN were prepared separately by setting the reaction times to 10 and 50 h, respectively. Subsequently, heat treatment was performed at 250 °C in hydrogen gas flowing at 1 L/min for 2 h to denitride the nitrided FeNi. The NITE process described above was performed as a series of operations without taking the specimens out of the electric furnace, except for cases where specimens are taken out for evaluation, to prevent the oxidation of specimens from exposure to the atmosphere. Once the NITE process was completed, the FeNi powder was taken out of the furnace and stored in ambient air.

### Characterization

The morphological observation of the FeNi powder was conducted using a field-emission scanning electron microscope FE-SEM (JSM-7100F) manufactured by JEOL. The specimens for SEM measurements were fabricated by affixing conductive double-sided tape on specimen holders and adhering FeNi powder directly on the adhesive surface. The crystal structure was evaluated using a powder X-ray diffractometer (SmartLab) manufactured by Rigaku. Fe-K_β_ (wavelength = 0.175653 nm) was used as the radiation source. The difference between the anomalous scattering of Fe and Ni with this wavelength was used to amplify the intensity of the extremely small superlattice diffraction lines of *L*1_0_-FeNi^22^. The derived regression patterns were compared against the patterns reported in the literature (ICSD Collection) or the simulation patterns provided by RIETAN-FP^23^. Literature data^24^ were referenced for the anomalous scattering factors of Fe and Ni used for simulations. High-angle annular dark-field imaging (HAADF) and element-mapping images were obtained using the atomic-resolution analytical STEM (JEM-ARM200F Dual-X) manufactured by JEOL. JED-2300 manufactured by JEOL was used as the EDX detector. STEM specimens were fabricated by procuring commercially available grids with supporting film; drops of alcohol containing FeNi particle powder diffused using an ultrasonic diffuser were applied to the grids, following which the alcohol was evaporated. The CG images of the crystal lattices were generated using VESTA^25^. Magnetometry was performed using a VSM (Versalab) manufactured by Quantum Design. Specimens for the VSM measurement were prepared by inserting pressurized powder in columnar shapes of 2 mm^Φ^ × 1.5 to 2 mm^h^ into measurement capsules. The filling rate of the pressurized powder was 20 Vol.%.

### Data availability

The authors declare that the data supporting the findings of this study are available within the article and its Supplementary Information files.

## Electronic supplementary material

Supplementary Figures and Tables
