# Supplementary material for: Synthesis of single-phase L10-FeNi magnet powder by nitrogen insertion and topotactic extraction
Source: Sci Rep. 2017 Oct 16;7:13216. doi: 10.1038/s41598-017-13562-2 (PMC5643398; doi:10.1038/s41598-017-13562-2)
Supplement: Supplementary file 1 — Supplementary Figures and Tables [file 41598_2017_13562_MOESM1_ESM.pdf]

Supplementary Figures and Tables

Synthesis of single-phase $L1_0$-FeNi magnet powder by nitrogen insertion and topotactic extraction

Sho Goto[1,*], Hiroaki Kura[1], Eiji Watanabe[1], Yasushi Hayashi[1], Hideto Yanagihara[2], Yusuke Shimada[3], Masaki Mizuguchi[3], Koki Takanashi[3], Eiji Kita[2,4]

[1]Research Laboratories, DENSO CORPORATION, Aichi 470-0111, Japan

[2]Institute of Applied Physics, University of Tsukuba, Ibaraki 305-8573, Japan

[3]Institute for Materials Research, Tohoku University, Sendai 980-8577, Japan

[4]National Institute of Technology, Ibaraki College, Ibaraki 312-8508, Japan

Correspondence and requests for materials should be addressed to S.G.

Supplementary Figures

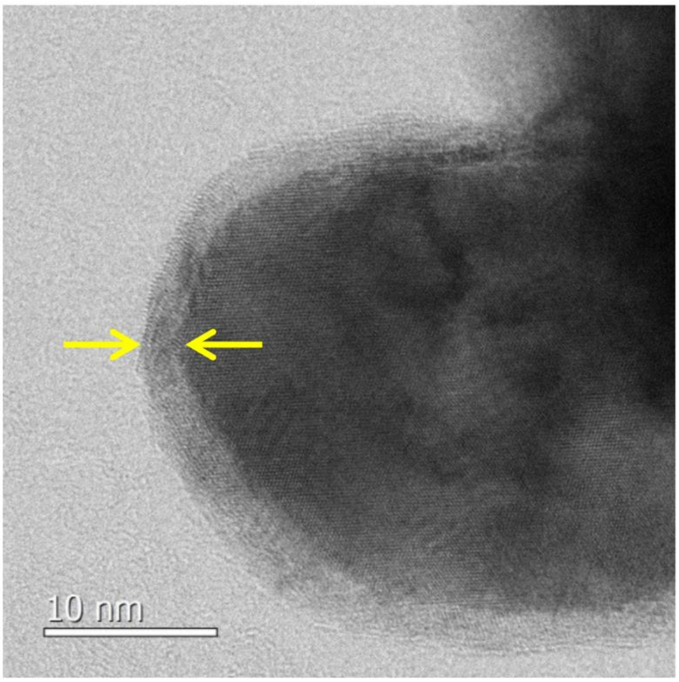

Supplementary Figure 1 TEM image of FeNi starting material

An oxide film was formed on the surface of the FeNi starting material. The thickness of the oxide film was approximately 2 nm.

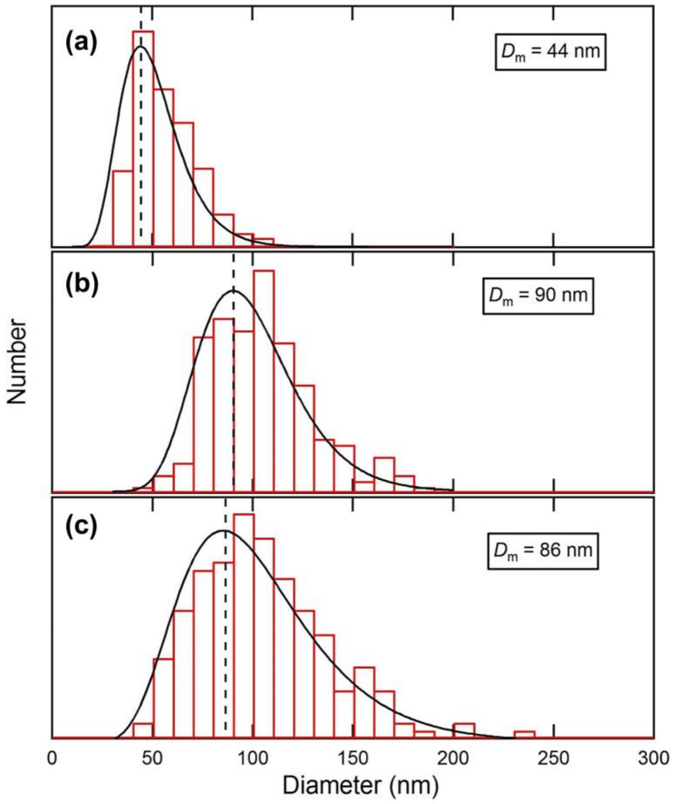

Supplementary Figure 2 Size distribution of (a) FeNi powder starting material, (b) FeNi powder after removal of oxidized film, and (c) FeNi powder after NITE processing. Each histogram was fitted by a log-normal function. Dotted lines indicate the mode diameters, D_m.

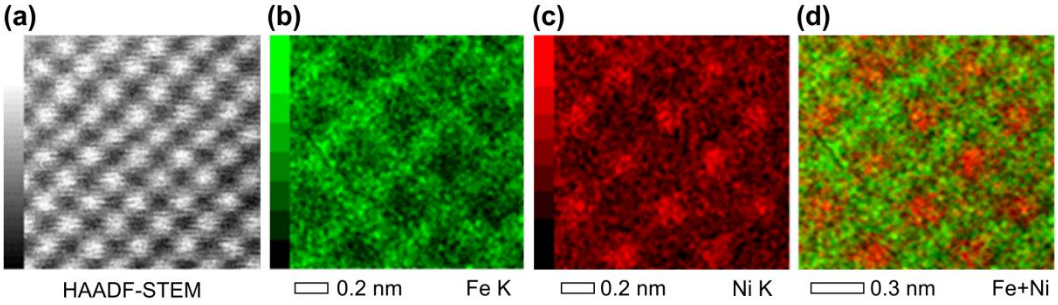

Supplementary Figure 3 HAADF images of DN-Fe_2Ni_2N, Fe element mapping, Ni element mapping, and element mapping overlay for Fe and Ni, observed from the <100> direction using the atomic-resolution analytical STEM. These images support the hypothesis that DN-Fe_2Ni_2N has an $L1_2$ structure in which corner sites are occupied by Ni atoms and face-centre sites are occupied by Fe and Ni atoms, as shown in Figure 1c.

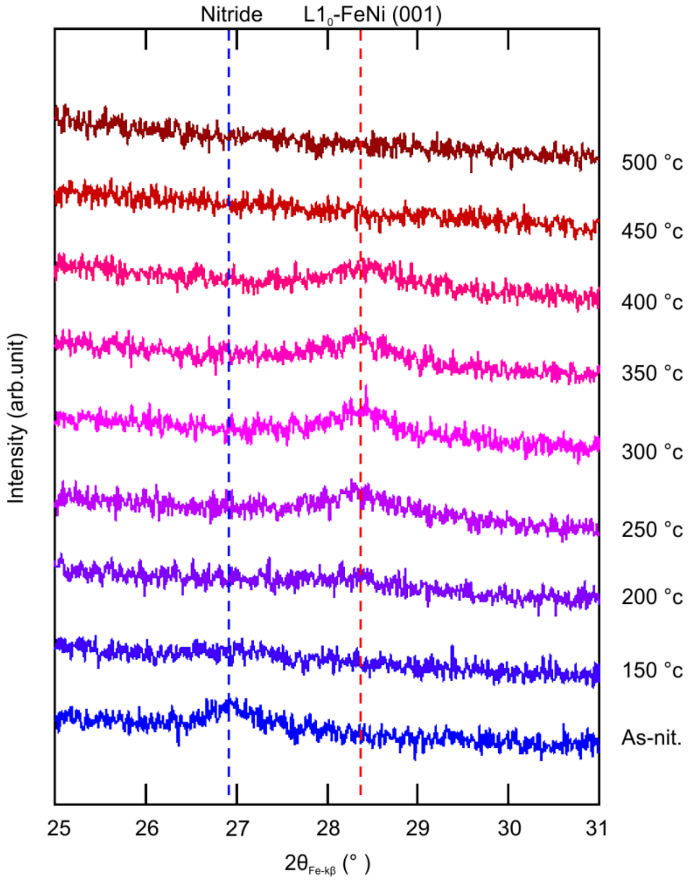

Supplementary Figure 4 XRD profiles of FeNi specimens denitrided at various temperature by annealing FeNi nitride in a H_2 atmosphere for 4 h. Superlattice peak of $L1_0$-FeNi disappeared at an annealing temperature greater than 450 ºC.

Supplementary Tables

Supplementary Table 1 Miller index (hkl), inter-planar spacing *d*, emergence angle 2θ, and normalized intensity of $L1_0$-FeNi with *S* = 1 and semi-ordered FeNi, as shown in Figure 1c. The values were calculated by RIETAN-FP under the assumption that both lattice constants are 0.358 nm and that the wavelength of X-rays was 0.1757 nm. Miller indexes with an asterisk indicate superlattice diffraction.

(hkl)	*d* (nm)	2θ (°)	Normalized intensity	
			$L1_0$-FeNi with *S* = 1	semi-ordered FeNi
(001)*	0.358	28.3	1.63	0.54
(110)*	0.253	40.6	1.58	0.53
(111)	0.207	50.2	100	100
(200)	0.179	58.7	48	48
(201)*	0.160	66.5	1.15	0.38
(112)*	0.146	73.8	0.93	0.31
(220)	0.127	87.8	29	29
(221)*	0.119	94.6	0.74	0.24
(301)*	0.113	101.6	0.05	0.02
(311)	0.108	108.8	34	34
